# User involvement and experiential knowledge in interprofessional rehabilitation: a grounded theory study

**DOI:** 10.1186/s12913-016-1808-5

**Published:** 2016-10-04

**Authors:** Mirela Slomic, Bjørg Christiansen, Helene L. Soberg, Unni Sveen

**Affiliations:** 1 Faculty of Health Sciences, Oslo and Akershus University College, Oslo, Norway; 2 Oslo University Hospital, Oslo, Norway

**Keywords:** User involvement, Patient involvement, Experiential knowledge, Traumatic brain injury, Multiple trauma, Interdisciplinary rehabilitation

## Abstract

**Background:**

User involvement is increasingly important in developing relevant health care services. The aim of this study was to contribute to a deeper understanding of user involvement and patients’ experiential knowledge as recognized and incorporated into clinical practice by rehabilitation professionals.

**Methods:**

A qualitative design using a grounded theory approach was applied. Data were collected by observations of the interprofessional meetings at two rehabilitation units treating patients with traumatic brain injury and multiple trauma and by individual semi-structured interviews with rehabilitation professionals.

**Results:**

The professionals recognized and incorporated user involvement into clinical practice as formal or authentic. Formal user involvement was sometimes considered pro forma. Incorporating patient’ experiential knowledge was considered a part of authentic user involvement. Possible gaps between the patients’ experiential knowledge and professional expertise were recognized. Challenges included dealing with ‘artifacts’, sources of information external to the patients’ own experiences, and addressing the patients’ possibly reduced insight due to trauma.

**Conclusion:**

Patients’ experiential knowledge was recognized as an essential component of the professionals’ knowledge base. The professionals considered user involvement and patients’ experiential knowledge as part of their clinical practice. Implementation of user involvement and contribution of patients’ experiential knowledge could be improved by understanding the issues raised in practice, such as possible negative consequences of user involvement in form of burdening or disempowering the patients. A better understanding of the characteristics and measures of user involvement is necessary in order to be able to offer its full benefits for both the patients and the professionals.

**Electronic supplementary material:**

The online version of this article (doi:10.1186/s12913-016-1808-5) contains supplementary material, which is available to authorized users.

## Background

User involvement is increasingly important in developing relevant health care services [1–3]. This development is partly a result of the New Public Management (NPM) and consumerism logic in the modernization of public health [1, 2]. Other important initiatives to ensure greater user involvement have originated in the disability rights movement and the democratic rights tradition [1, 2, 4]. Individual user involvement is aimed at strengthening each individual’s rights and possibility of choices. In addition to individual user involvement, representative or public user involvement is aimed at giving opportunity for the public to voice their opinion on and shape development of health care services [5]. In Norway, both levels of user involvement are granted by law [4]. In this article, the term user involvement encompasses both individual patient involvement and representative user involvement.

In practice, user involvement does not necessarily lead to better quality or provision of care, and it can be both empowering and disempowering [1]. Self-management training programs aiming to create an expert patient were accepted early on and are still partly the dominant model of user involvement [3]. However, this approach to user involvement is criticized as being overly simplistic [3]. The current focus is on the need for a broader approach to user involvement that also comprises the family, social and political contexts [3, 6]. User involvement in health care shares a number of positive characteristics with patient-centered care as well as its challenges and barriers, [7, 8] as they both have the common focus of including patients in their own care.

Evidence-based practice (EBP), in addition to the best research evidence, must include professional expertise and patients’ experiential knowledge [9]. Good EBP is expected to consider these factors and extend beyond patients’ choices [10]. More attention should be paid to the biopsychosocial contexts of each respective patient [11].

Professionals conceptualize user involvement as a potentially beneficial process that is a dynamic rather than a static feature of clinical practice [12], whereas patients’ experiential knowledge is valued because it may improve care delivery [13]. How user involvement and experiential knowledge intersect with professional expertise in clinical settings is insufficiently understood. Throughout this article, we used the term patients’ experiential knowledge, where we refer to the knowledge the patients have about their own lived experiences. The chosen topic of the present study is the interprofessional rehabilitation of patients with traumatic brain injury (TBI) and multiple trauma. These patients experience reduced physical, psychological and social function and reduced health-related quality of life [14, 15]. Disability caused by TBI and multiple trauma affects everyday life and social and vocational participation [14, 16]. Mental health problems [17], cognitive disability and effects on self-awareness [18, 19] can also occur, possibly leading to additional challenges in the implementation of user involvement in practice.

The aim of this study is to contribute to a deeper understanding of how user involvement and patients’ experiential knowledge are recognized and incorporated into clinical practice by the professionals working in interprofessional rehabilitation.

## Methods

This study had a qualitative design using a grounded theory approach [20]. The comparative and interactive nature of this approach offered the possibility to simultaneously analyze and gather new data in order to answer emerging empirical questions. Gathering of consecutive data was informed by previously gathered data and an ongoing data analysis. Constructivist grounded theory [20], applied in this study, allowed for contextual understanding, while we attempted to avoid theoretical preconceptions.

The study was based on observations of eight meetings of interprofessional teams at two specialized rehabilitation units in southeastern Norway and on semi-structured in-depth interviews with 16 participating rehabilitation professionals. The observations and the interviews were conducted between April 2014 and April 2015.

The study was part of a larger project called ‘Transitions in Rehabilitation’ that explored different aspects of rehabilitation of patients with TBI and multiple trauma. The project also entailed a user panel with representatives from relevant user organizations. The representatives have personal experiences either as patients with TBI or multiple trauma themselves or as next of kin.

### Setting

The data were collected at one rehabilitation unit that admitted inpatients and one outpatient unit. At both units, the interprofessional meetings focused on patients’ goals, progress and plans regarding their rehabilitation process and were conducted at least once a week. Typical participants in the meetings were medical doctors, nurses, occupational therapists, physical therapists, psychologists, social workers, special educators and team coordinators. Individual patients participated in some of the meetings. Patients’ participation in the meeting was intended to strengthen user involvement and was organized in such a way that one patient participated in the meeting related to that particular patient’s rehabilitation process. The meetings with the participating patients were conducted several times during the patient’s stay—usually after admittance, halfway during the planned stay and prior to discharge. Written individual rehabilitation plans were used to ensure user involvement and progress towards common goals that the patients and the participating professionals had discussed and agreed upon.

### Participants

Purposive sampling was used in this study, which allowed for flexibility in the sampling strategies throughout the research process [20, 21]. The professionals selected were working with TBI and multiple trauma patients. In the eight observed meetings, 41 individual professionals participated, including three students (two physiotherapy and one psychology). The patients participated in four of the eight observed meetings. The number of participants in the meetings varied from two to 14 professionals. Informants were selected based on observations of the meetings and the participants’ activity during the meetings. The rehabilitation professionals either responsible for the patients discussed, or those who contributed extensively to decision-making during the observed meetings were selected for individual interviews. Individual semi-structured interviews were conducted with 16 individual professionals: one medical doctor, two nurses, three occupational therapists, two physiotherapists, three psychologists, two social workers, one special educator and two team coordinators.

### Data collection methods

The observations of the eight interprofessional meetings focused on interactions, patterns of communication and decision-making, and patient involvement either as active participants in the meetings or as voiced by the participating professionals. The observations offered the possibility to observe the context, routines and practices that the participants themselves might take for granted [22]. Notes were taken during the observations, which informed the interviews and were used during the data analysis process. The meetings were audio-recorded and transcribed verbatim.

The individual interviews were conducted after the observed meetings with 16 of the participating professionals at their work place. Due to the professionals’ busy schedules, the interviews were limited to a maximum of 45 min, ranging from 20 to 45 min, with an average of 26 min. The interview guide (Additional file 1) was semi-structured with seven discussion topics and suggested open-ended questions aimed at uncovering the professionals’ experiences, perspectives, motives and attitudes regarding their interprofessional rehabilitation practice [21, 23]. For this current study in particular, the focus was on how users’ perspectives and user involvement were incorporated into the rehabilitation process. Particular attention was paid to the position that the professionals ascribed to the patients’ experiential knowledge in the hierarchy of knowledge used for decision-making in practice. The interviews were audio-recorded and transcribed verbatim. Data collection was terminated when theoretical saturation was reached and no new topics emerged during the observations and the interviews.

### Data analysis

Data analysis was based on a grounded theory approach [20]. Data analysis began during the observations, as the interviews were planned and the interview participants selected during the observations.

The first transcripts were read to gain a sense of the whole, and the initial codes were coded by hand and discussed among the authors. Subsequently, all transcripts were coded using HyperResearch software tool (ResearchWare, Inc., Randolph, MA, USA). The codes were condensed and the emerging categories were identified (Table 1) and confirmed using the grounded theory approach of constant comparison with the transcripts [20].

**Table 1:** Example of data analysis in the study

Data	Initial codes	Categories	Main category
‘One works actively with the patient, each of the team members talks with the patient about the goals, but ultimately it is the patient who decides…’	Working personally with the patient	Personal contact with the patient	Authentic user involvement
	Talking with the patient		
	Relating to the patient’s goals	Understanding the patient’s preferences	
	Prioritizing the patient’s wishes		

Analytic categories were identified, leading to a common model for understanding how the professionals viewed and incorporated user involvement and patients’ experiential knowledge in interprofessional rehabilitation.

## Results

The rehabilitation professionals recognized and incorporated two forms of user involvement in their clinical practice, which we termed formal and authentic user involvement. Incorporating patients’ experiential knowledge in the rehabilitation assessment was considered part of authentic user involvement. The professionals identified several domains in which gaps between the patients’ experiential knowledge and professional expertise could occur.

### Formal user involvement

Formal user involvement, according to the professionals, included patients’ informed consent throughout their rehabilitation process, patient participation in the interprofessional meetings and activities involving the user organizations. Although formal user involvement was fulfilled, the professionals did not always regard this formal involvement as appropriate or relevant but rather as pro forma. The professionals expressed the belief that patient participation in interprofessional meetings could be perceived as an additional burden for the patients rather than a means of having their voices heard.

*‘I think that sometimes it might be hard for the patients to come up with their perspective in those meetings [interprofessional meetings with participating patients].’* (Psychologist—interview)

Rather than empowering the patients in taking part in the decision-making regarding their rehabilitation, the meetings could have the opposite effect of disempowering the patients.

The observed meetings in which the patients participated had less discussion among the professionals than the meetings without the patients. The professionals represented a united point of view in discussions when the patients participated in the meetings. This was partly justified in caring for the patients and not introducing additional confusion.

*‘If there are exceptional things that we have to discuss, that the team must agree on, we discuss it among us… that is to say without the patient, and that way we can discuss it a bit more freely and disagree to some extent… so we do not do that in front of the patient.*’ (Social worker—interview)

Although the professionals attempted to include the patients in the meetings, the patients’ active involvement varied. The variation could be explained in part by the meetings being organized according to premises specific to the particular settings of the rehabilitation units included in this study. Additional challenges in implementing formal user involvement in practice were recognized by the professionals, who considered that a patient might lack insight or knowledge about their expected role in goal setting or decision-making.

Written rehabilitation plans served as a cooperation tool during the meetings, but also outside of the meetings. The patients had access to their own rehabilitation plan, and were involved in developing it together with the professionals. The rehabilitation plans included information about specific goals, time frames for reaching those, and professionals responsible for supporting the patient during this process. Usefulness of the rehabilitation plans was related to development of comprehensive cooperation both among the rehabilitation professionals and between the professionals and the patients. Using written individual rehabilitation plans was considered useful but not an adequate reflection of patients’ actual goals for interprofessional rehabilitation.

*‘One works towards the goals written there [in the patient’s rehabilitation plan]… towards the patient’s overarching goals that are much more long-term and sometimes not very realistic, but it must be the patient’s own goals, which are modified along the way… Then there are sub-goals… that we help the patient set up because… we try to work towards something called SMART [specific, measurable, achievable, realistic/relevant and timely goals].’* (Psychologist—interview)

Another issue was the involvement of user representatives and user organizations in clinical settings regarding daily clinical practice. The professional sometimes considered this representative involvement to be irrelevant when dealing with the individual patients, both when considering local user representatives at specialized rehabilitation units or branches from larger user organizations.

*‘We considered it [having user representatives] but did not find the completely right forum for it… so user involvement is primarily what happens in the follow-up of an individual.’* (Social worker, interview)

The value of this representative level of user involvement was attributed to the organizational or system level rather than to everyday clinical practice. The professionals considered representative user voice relevant in developing health care services in general, but they did not consider it relevant in an individual patient follow-up.

### Authentic user involvement

According to the professionals, authentic user involvement was expressed through the daily contact and interaction between patients and professionals, in daily conversations, personal involvement, and in referencing the patients’ needs and plans in cooperation with other professionals. The patient was thought of as a unique individual within a complex contextual setting. The professionals conveyed the importance of a contextual approach by referring to the patients’ life situation during the meetings.

*‘There is more than the usual burden at home; one of her children who does not live at home is still often there with [diagnosis] that is… quite challenging at times… Another child that does live at home…There is also something about the level of performance that the patient shows at home.*’ (Occupational therapist—observed meeting)

The focus of the meetings was not only on the patients’ medical condition but also on their family, work and social setting in general. Goal-setting strategies and the patients’ progress in their rehabilitation process were adjusted according to the biopsychosocial approach as well. Approaching the patient holistically was viewed as a way of tailoring the rehabilitation care to each individual patient.

*‘We are concerned about the home situation, the work situation and the circumstances surrounding the patient outside of the institution.’* (Team coordinator—interview)

In the professionals’ opinion, the best way to involve the patients in their own rehabilitation process included recognizing patients’ wishes, setting goals and keeping track of progress according to the patients’ preferences. This approach was also considered to lead to patient empowerment, as they became more active in their own rehabilitation process. The optimal method to achieve this authentic type of user involvement was considered to be daily contact with and having personal knowledge of the patient.

*‘I am concerned with getting an understanding of who they [the patients] are personally; what life they lead and what the person him/herself experiences as changed after what happened.’* (Occupational therapist—interview)

The professionals perceived authentic user involvement as an optimal form of user involvement and as a time-consuming process. Involving inpatients in their own rehabilitation was perceived as a more straightforward process compared with involving the outpatients.

*I see it [working with inpatients over time] as an advantage because one comes to know each other and…one contributes to making their [the patients’] everyday life as good as possible.’* (Nurse—interview)

The rationale behind perceiving the involvement of the inpatients as a simpler task was based on the continuity of personal contact and the knowledge of the patients as they grasped their own rehabilitation process.

### Patients’ experiential knowledge

The rehabilitation professionals considered patients’ experiential knowledge to be unique and to offer a range of opportunities for improving patient-centered rehabilitation and daily clinical routines. The unique value of the patients’ experiential knowledge was that it was based on their own life experiences, needs, wishes and possibilities, which bound the patients’ experiential knowledge to user involvement.

*‘At the same time, the experience we have primarily comes from other patients… To fully understand a patient with a brain injury, we will always be somewhat limited unless we inflict a head injury on ourselves to see how it is experienced first-hand.’* (Psychologist—interview)

It was necessary to incorporate patients’ unique experiential knowledge into daily clinical practice as part of evidence-based practice and the biopsychosocial approach. This approach could include a transformation of the patients’ experiential knowledge and lead to a common understanding of rehabilitation and the rehabilitation process between the patients and the professionals.

Incorporating patients’ experiential knowledge elicited a range of challenges with possible gaps between the patients’ experiential knowledge and the professionals’ expertise. Parts of the patients’ knowledge were considered artifacts if they did not originate from the patients’ own lived experiences but rather were from external sources, such as various unvalidated online forums or social networks. The professionals expressed their concerns about the challenges posed by patients’ artifact-knowledge primarily in the interprofessional meetings in which the patients were not present. Categorical rejection of such knowledge was expressed during those meetings, intended to prevent the patient from being misguided in their rehabilitation process.

*‘But when you [the patient] start reading on those websites… there I think you have lost your way.’* (Occupational therapist - meeting)

*‘Yes, the patient read those forums…and that is a big black hole, right?’* (Medical doctor—meeting)

The professionals conveyed a high value to the validation of information and knowledge sources. While continuing the discussion about the patient ‘*losing his/her way*’ by reading certain websites, a participating physiotherapist commented that no one was checking the sources that were referred to on the mentioned website. However, the professionals understood the patients’ need to search for additional sources of information and knowledge about their conditions. Hence, the professionals suggested alternatives to the professionally or scientifically questionable sources.

A further challenge was the possible lack of insight, particularly in the patients with TBI. According to the professionals, the patients did not always have the necessary understanding of the system and the rehabilitation services offered at the rehabilitation unit.

*‘Sometimes the patients have unrealistic expectations about the available services and our responsibilities, so it can be ok to guide them a bit.’* (Physiotherapist—interview)

The professionals considered it a part of their professional role to orient the patients by addressing the gaps between patients’ experiential knowledge and their professional expertise.

*‘The patients have their own needs and interpretations that we have to reinterpret and translate into the patient’s best interest when we come up with suggestions to the patient.*’ (Medical doctor—interview)

The basis for bridging the possible gaps between the patients’ experiential knowledge and professional expertise was considered to be understanding and respecting the patients’ experiential knowledge while attuning it to professional knowledge and the available services.

## Discussion

This study found that user involvement was incorporated into clinical practice either as authentic or formal user involvement. The professionals regarded formal user involvement at times to be pro forma and the representative user involvement to be detached from the individual patient’s care. Patients’ experiential knowledge was considered a part of authentic user involvement by the rehabilitation professionals.

The current study found that user involvement was an integral part of daily clinical rehabilitation practice. The rehabilitation professionals viewed authentic user involvement as a form of user involvement that granted patients an individual voice and choice in practice. The professionals acknowledged representative user involvement as a means of influencing the services offered [5]. Rather than being perceived as authentic user involvement, the representative user involvement was acknowledged as providing possibilities for influencing the service development in such a way that enables the professionals to embrace authentic user involvement in daily clinical practice. Here, individual user involvement represented the main attempts to strengthen each patient’s rights and choices [1]. At the rehabilitation units included in this study, the professionals mainly focused on two modes of individual user involvement—authentic and formal. Representative user involvement was not considered to be specifically important when working with the individual patients. User representatives were not considered active partners in the individual patients’ rehabilitation process, as they did not have influence on discussions and decision-making regarding the individual patients.

Authentic user involvement as described by the professionals had an empowering function, whereas formal user involvement could be disempowering or even burdening to the patients. Dent and Pahor [1] described user involvement as both empowering and disempowering for each of the following patient types: patients as consumers, participants and citizens. Similarly, in this study, possible negative consequences of user involvement were described regarding user involvement that the professionals considered pro forma. The present study points to the need to create formal user involvement in the rehabilitation process that serves to strengthen patients’ roles in rehabilitation practice and that becomes transformed into authentic user involvement. Although the professionals acknowledged the possible benefits of including the patients in the interprofessional meetings, they recognized the challenges in consistently implementing successful user involvement in this setting.

The professionals extensively discussed user involvement as related to setting individual rehabilitation goals according to the individual patients’ needs, plans and preferences. To ensure user involvement in goal-setting situations, written rehabilitation plans were used. The plans were considered valuable tools both when working with the patients and among other professionals. However, professionals sometimes perceived these documents as inadequate in reflecting the patients’ own rehabilitation goals. This could be due to fundamental differences in the different positions and understandings of the patients’ and the professionals’ goals, as has been reported in previous research [24–27]. Suganavam [27] also described that although studies on the effects of and experiences with goal setting reported some positive outcomes, the extent of user involvement in goal setting remained unclear.

In the observed meetings, the level of patient-centeredness varied regarding the individual patient’s condition and their specific contextual situation. Similar findings have been reported previously [24, 27]. The professionals in the present study also expressed concern that the patients insufficiently understood their role in goal setting and participating in the interprofessional meetings. However, the professionals partly described their own responsibility to give the patients a voice both during the meetings and particularly in their personal contact with the patients. Relating the patients’ personal goals to the rehabilitation goals was considered essential. The professionals attempted to attune the patients’ wishes, needs and goals to the rehabilitation services offered. They also assisted the patients in formulating goals. Lequerica et al. [28], who reported that making therapy tasks meaningful and explicitly related to personal goals was a common practice for enhancing therapeutic engagement, have reported similar approaches by other professionals. The present study showed that professionals were aware of the necessity to relate patients’ rehabilitation process to their overall goals and life. The professionals’ concern for who the patients were before the injury and for the broader context of the patients’ life and living was related to this necessity. The participants in this study termed this approach as holistic. Sivaranam [29] also described a need for concurrence between patients’ life goals and treatment goals.

Formal user involvement was not automatically characterized as pro forma, but rather additional actions had to be taken to transform it into authentic user involvement. In order for formal user involvement to become authentic, it had to offer each individual patient actual possibilities of being understood, and opportunities to influence his/her own rehabilitation process. Formal individual user involvement, such as the previously discussed participation in the interprofessional meetings and using individual rehabilitation plans, had only limited value. If this approach was to be regarded as authentic user involvement, the patients had to understand fully their role when participating in either the meetings or development of their own rehabilitation plans. Additionally, the professionals had to support the patient in gaining this necessary understanding, while under tight schedules of clinical work. Therefore, authentic user involvement according to professionals was actually developed in the daily patient contact by understanding and acknowledging the patients as unique individuals in unique contextual settings, rather than through formalizing user involvement in itself.

Possible gaps between patients’ experiential knowledge and professional expertise were recognized. Conflict may arise between professionals’ and users’ perspectives on the rehabilitation process or because of the patients’ reduced awareness. The professionals perceived incorporating patients’ experiential knowledge into their rehabilitation practice as a means to bridge the possible gaps between patients’ experiential knowledge and their own expertise. Overcoming the possible gaps led to a common understanding of the rehabilitation process between the patients and professionals. The patients’ experiential knowledge had an impact on decision-making in interprofessional rehabilitation and was considered a part of authentic user involvement. In this regard, involving the users meant involving the users’ experiences and knowledge based on their lived experiences prior to, during and throughout their own rehabilitation process.

The professionals’ interpretations of the patients’ experiential knowledge could sometimes be viewed as paternalistic, but they mostly considered the patients’ experiences and ‘expertise’ while adjusting it to suit their clinical needs. Addressing the patients accordingly could be considered a mild form of paternalism. This form of paternalism could be considered beneficial if it is aimed at strengthening the patients’ autonomy [30]. The professionals attempted to strengthen the patients’ autonomy and opportunities for decision-making by offering additional information about the available services and the rehabilitation process.

### Strengths and limitations

One strength of this study was the method of data collection, with complementing observations and individual interviews. The professionals interviewed in the study were another strength, as informants from each of the professional groups involved in rehabilitation were interviewed. A possible limitation might be limiting the data collection to only two rehabilitation units. However, they were considered representative of specialized rehabilitation units in general and were strengthened by including an inpatient and outpatient rehabilitation unit. The relatively short time for some of the interviews might be a limitation of this study. However, our goal was theoretical saturation. Therefore, and due to extreme scarcity of new information during the last two interviews, we considered the 16 conducted interviews representative for understanding the process of incorporating user involvement and patients’ experiential knowledge in rehabilitation practice in this setting. Another possible limitation was not including users as informants when researching user involvement and experiential knowledge. However, this particular study was focused on the professionals’ perspectives, as another part of the overarching ‘Transitions in rehabilitation’ project includes the patients’ perspectives.

A strength of the data analysis of this current study was the multi-professional background of the research team, comprising medicine, nursing, physical therapy and occupational therapy, which strengthened the validity and reliability of the results. Additionally, testing the emerging results during the analysis strengthened the validity. One additional strength was the discussions within the user panel, which offered valuable insight into the topics covered during the observations and the interviews, provided useful input during the data analysis process and discussion of the results, and provided additional validation to the study’s findings.

## Conclusion

Patients’ experiential knowledge was recognized as an essential component of the professionals’ knowledge base. The professionals considered user involvement and the patients’ experiential knowledge as part of their clinical practice. They related the patients’ experiential knowledge to authentic user involvement, which they considered to be understanding and acknowledging the patients as unique individuals in unique contextual settings. Formal user involvement was considered pro forma where it did not enable or encourage the individualized approach to the patients, and was rather perceived as ‘going through the motions’.

Understanding the implications and consequences of user involvement in practice is an important aspect of improving current practices. Professional’s experiences regarding user involvement and patients’ experiential knowledge should be considered in policy-making and in practical implementation of user involvement in clinical settings. Attention should be paid to understanding authentic user involvement. Implementation of user involvement and contribution of patients’ experiential knowledge in rehabilitation could be improved by understanding the issues raised in practice, such as possible negative consequences of user involvement in form of burdening or disempowering the patients.

Additional research is needed in order to confirm the importance of authentic user involvement both for the patients and the professionals, as well as to confirm the applicability of concept in other clinical and geographical areas. A better understanding of characteristics and measures of authentic user involvement is necessary in order to be able to provide its full benefits for both the patients and the professionals.

## Additional file

Additional file 1: Interview guide. (DOCX 13 kb)

## Abbreviations

EBP: Evidence-based practice
NPM: New Public Management
TBI: Traumatic brain injury
