# Supplementary material for: User involvement and experiential knowledge in interprofessional rehabilitation: a grounded theory study
Source: BMC Health Serv Res. 2016 Oct 4;16:547. doi: 10.1186/s12913-016-1808-5 (PMC5051024; doi:10.1186/s12913-016-1808-5)
Supplement: Additional file 1: — Interview guide. (DOCX 13 kb) [file 12913_2016_1808_MOESM1_ESM.docx]

***Additional file 1: Interview guide***

Interview topics/questionnaire

The following topics will be addressed in the interviews. The questionnaire is meant to serve as a simple guidance and a great degree of freedom in interviewing will be practiced. However, the idea is to cover same topics in all interviews, as to have a certain degree of comparability.

- Background: educational/professional background, time working within (interprofessional) rehabilitation:
  - What is your educational and professional background?
  - How long have you worked within rehabilitation?
- One’s own role in interprofessional collaboration:
  - How would you describe your role in interprofessional rehabilitation?
  - Do you feel that you were listened to/that your opinion was valued in this meeting?
  - How were your assessments taken into account/accepted by other participants in the meeting?
- Interprofessional collaboration and interprofessional meetings:
  - What is your opinion on working in interprofessional collaboration in general?
  - Please describe a typical/usual interprofessional meeting!
  - What is your opinion on structure and organization of interprofessional meetings?
  - What do you think works best in an interprofessional meeting?
  - Is there something that could be different/organized differently?
  - How was this particular meeting different from typical/usual meetings (if at all different)?
  - How was interprofessional collaboration in this particular meeting?
  - How were different inputs presented in the meeting?
  - What was the most important topic in the meeting?
  - What was behind the decisions taken in the meeting?
  - Was there some input that was not followed up (why)?
- Collaboration partners/different professions and their role in rehabilitation process:
  - Whom do you think it is usually most valuable to listen to either in interprofessional meetings or collaboration work?
  - Is there somebody’s assessment that you value most (whose)?
- Documentation: e.g. patient journal, individual plan and similar:
  - How would you describe the use of patient journals in interprofessional collaboration?
  - Can you describe using individual plan or similar documentation in interprofessional collaboration?
  - Please describe the impact of professional guidelines and classifications on interprofessional collaboration!
- Patients’ role – either as active participants in the meeting or voiced through participating professionals:
  - How was the patient’s role taken care of in the meeting?
  - Can you describe some of the issues in addressing the patients’ perspectives throughout rehabilitation processes?
  - How are possible challenges solved by including the patients’ perspectives in professional work?
- Municipal and work-related actors/employers’ role in the rehabilitation process:
  - Please describe the cooperation with municipal rehabilitation units!
  - What is your opinion on work-related issues and employers’ role in rehabilitation processes?
  - How can specialized rehabilitation services facilitate transition to local care and getting back to work/other activities?
- Would you like to add something or explain something that we did not cover during the interview?
